# Two New Cytotoxic Candidaspongiolides from an Indonesian Sponge

**DOI:** 10.5402/2011/852619

**Published:** 2011-07-18

**Authors:** Agus Trianto, Idam Hermawan, Toshimasa Suzuka, Junichi Tanaka

**Affiliations:** ^1^Department of Chemistry, Biology, and Marine Science, University of the Ryukyus, Senbaru 1, Nishihara, Okinawa 903-0213, Japan; ^2^Department of Marine Sciences, Faculty of Fisheries and Marine Sciences, University of Diponegoro, Semarang, Central Java 50234, Indonesia

## Abstract

Marine sponges have been recognized as potentially rich sources of various bioactive molecules. In our continuing search for new secondary metabolites from Indonesian marine invertebrates, we collected a sponge, whose extract showed cytotoxicity against cultured cells at 0.1 *μ*g/mL. Purification of the extract yielded two new macrolides **2** and **3** along with known candidaspongiolide (**1**). The structures for compounds **2** and **3** were elucidated by spectral analysis (^1^H, ^13^C, COSY, HMQC, HMBC) and by comparison of their NMR data with those of **1**. Compounds **2** and **3** exhibited a little more potent cytotoxicity (IC_50_ 4.7 and 19 ng/mL) than that (IC_50_ 37 ng/mL) of candidaspongiolide (**1**) against NBT-T2 cells.

## 1. Introduction

Sponges, a group of sedentary organisms, cannot move and escape from predators. Most sponges are filter feeders pumping water to its body to obtain foods and oxygen and to expel wastes and may be threatened by microorganisms during filtering seawater rich in bacteria and fungi [1, 2]. In order to defend themselves against predators, pathogens and competitors, sponges may have developed to produce or accumulate secondary metabolites during their long evolution, such as feeding deterrent, antimicrobial, antifungal, and antifouling molecules. Interestingly, some of the compounds have also shown remarkable potency as drug candidates against various human diseases as discussed elsewhere [3–9]. 

In 1984, Schmitz and coworkers isolated tedanolide from the Caribbean marine sponge *Tedania ignis* [10]. Tedanolide is a unique 18-membered macrolide where lactonization occurs at a primary hydroxyl group instead of a common secondary one, and this class of macrolide has been reported to exhibit strong cytotoxicity at pico to nanomolar range [10, 11]. The unique structure in combination with promising biological activity leads tedanolide as an intriguing target for formal and total syntheses [12–14]. More recently, Meragelman and coworkers reported a macrolide named candidaspongiolide (**1**) related to tedanolide with modification at C-11 to C-15 from the marine sponge *Candidaspongia* sp. Candidaspongiolide exhibited potent cytotoxicity in NCI 60 cells panel with GI_50_ of 14 ng/mL [15], protein synthesis inhibition, and apoptosis induction [16]. 

In our continuing search for potential drug leads from Indonesian marine invertebrates [17, 18], we obtained a sponge whose extract showed cytotoxicity at 0.1 *μ*g/mL against NBT-T2 cells in a screening process. Purification of the extract provided candidaspongiolide (**1**) along with two new analogs **2 **and **3**, which are the subject of this paper.

## 2. Materials and Methods

### 2.1. Chemicals and Equipments

Methanol (MeOH) used for extraction was of technical grade. Reagent grade solvents were used for isolating compounds **1**–**3**. Merck Si-60 (70–230 mesh) was used for silica gel column chromatography, while Merck Si-60 F_254_ for analytical TLC. HPLC was performed either on a Waters 510 pump with a Waters 486 UV detector and a Shodex RI-101 or on a Hitachi L-6000 pump with a Hitachi L-4000 UV detector and a Shodex RI-101 using a Mightysil Si-60 (10 × 250 mm) column. Optical rotations were measured on a Jasco P-1010 polarimeter using a cell with 3.5 mm aperture. IR spectra were recorded on a Jasco FT/IR-6100 instrument, whereas HRESIMS was measured on a Jeol JMS-T100LP spectrometer using reserpine or sodium trifluoroacetate as an internal standard. Most of ^1^H and ^13^C NMR spectra were measured in CDCl_3,_ while those of compound **3** were measured in CD_3_OD with TMS as an internal standard on a Jeol A500 and/or a Bruker AVANCE III-500 in CDCl_3_. The ^1^H and ^13^C chemical shifts were given in ppm, while coupling constants were in Hz.

### 2.2. Sponge

Specimens of the sponge tagged K09-02 was collected by hand using SCUBA at 15–25 m depth at Kupang, West Timor, East Nusa Tenggara, Indonesia on August 2009. By comparing underwater images of our specimen with that of the specimen of NCI group [15], it is likely to be the same sponge. The specimen was kept frozen until extraction. The sponge K09-02 may be an endemic species to this region. The colonies are grey in color and stand.

### 2.3. Extraction and Isolation

After cutting into small pieces, the sponge (653 g, wet) was soaked in MeOH for 24 h for three times. Then, the solution was concentrated under vacuum to obtain a crude extract. The methanolic extract (17.0 g) was triturated with ethyl acetate (EtOAc) to provide a lipophilic fraction (2.7 g), which killed NBT-T2 cells at 0.1 *μ*g/mL. This fraction was subjected to a silica gel column eluting with stepwise gradient solvents (hexane : EtOAc = 2 : 1, 1 : 1, 1 : 2, 0 : 1, EtOAc : MeOH = 10 : 1) to afford ten fractions. Fraction 5 (126.0 mg) was purified by repetitive Si-60 HPLC using hexane-EtOAc mixtures to provide candidaspongiolide **1** (15.4 mg). Fraction 6 (70.8 mg) was also purified by Si-60 HPLC using a solvent system hexane : EtOAc = 2 : 1 to afford compound **2 **(9.8 mg). Fraction 9 (107.1 mg) was also subjected to repetitive Si-60 HPLC using hexane : EtOAc = 1 : 6, EtOAc : CH_2_Cl_2_ : MeOH = 20 : 20 : 1, and EtOAc : CH_2_Cl_2_ : MeOH = 10 : 20 : 1 as solvent systems sequentially, to give compound **3 **(21.8 mg). Isolation of these compounds was guided by cytotoxicity testing and NMR spectra.

### 2.4. Compound **1**

Colorless glass, [*α*]_D_^25^ +69 (*c* 0.55, MeOH). IR *ν*_max⁡_ (neat) 3419, 2925, 2854, 1742, 1715, 1456, 1372, 1234, 1086 cm^−1^. ^1^H and ^13^C NMR; see Tables 1 and 2. HR-ESIMS [M+Na]^+^ *m*/*z* 945.55514, 959.57079, 973.58884 (calcd for C_50_H_82_NaO_15_^+^ 945.55459 (Δ +0.58 ppm), C_51_H_84_NaO_15_^+^ 959.57024 (+0.57 ppm), and C_52_H_86_NaO_15_^+^ 973.58589 (+3.0 ppm)).

### 2.5. Compound **2**

Colorless glass, [*α*]_D_^25^ +72 (*c* 0.75, MeOH). IR *ν*_max⁡_ (neat) 3421, 2925, 2854, 1748, 1715, 1456 cm^−1^. ^1^H and ^13^C NMR; see Tables 1 and 2. HR-ESIMS [M+Na]^+^ *m*/*z* 903.54964, 917.56023, 931.57576, 945.60036 and 959.61056 (calcd for C_48_H_80_NaO_14_^+^ 903.54403 (Δ +6.2 ppm), C_49_H_82_NaO_14_^+^ 917.55968 (+0.60 ppm), C_50_H_84_NaO_14_^+^ 931.57533 (+0.46 ppm), C_51_H_86_NaO_14_^+^ 945.59098 (+9.9 ppm), and C_52_H_88_NaO_14_^+^ 959.60663 (+4.1 ppm)).

### 2.6. Compound **3**

Yellow glass, [*α*]_D_^25^ +97 (*c* 0.35, MeOH). IR *ν*_max⁡_ (neat) 3418, 2925, 2854, 1715, 1457, 1373, 1244, 1084, 995 cm^−1^. ^1^H and ^13^C NMR; see Tables 1 and 2. HR-ESIMS [M+Na]^+^ *m*/*z* 665.31522 (calcd for C_32_H_50_NaO_13_^+^ 665.31436 (+1.3 ppm)).

### 2.7. Acetylation

Compound **1** (0.2 mg) was dissolved in pyridine (50 *μ*L) and acetic anhydride (50 *μ*L). The mixture was stirred for three days under a nitrogen atmosphere at room temperature. After removal of excess reagents with nitrogen flow and vacuum, the reaction product **4** was checked with ^1^H NMR and ESIMS. Compound **2** was similarly treated to give **4**.

### 2.8. Compound **4**   from   **1**

^1^H NMR: *δ* 5.65 dd (*J* = 2.7, 9.5 Hz), 5.52 m, 5.51 d (*J* = 10.1 Hz), 5.36 m, 5.34 dd (*J* = 2.2, 9.1 Hz), 5.28 dt (*J* = 2.3, 10.5 Hz), 5.07 d (*J* = 11.0 Hz), 4.79 d (*J* = 6.6 Hz), 4.54 dd (*J* = 2.0, 11.3 Hz), 4.25 dd (*J* = 2.3, 8.1 Hz), 4.13 dd (*J* = 6.3, 11.5 Hz), 3.43 s (3H), 3.34 dq (*J* = 10.0, 6.7 Hz), 3.29 dq (*J* = 10.5, 7.0 Hz), 3.01 dd (*J* = 9.2, 18.4 Hz), 2.88 d (*J* = 9.3 Hz), 2.60 dd (*J* = 2.6, 18.4 Hz), 2.35–2.4 m, 2.32 dd (*J* = 1.9, 9.3 Hz), 2.21 s (3H), 2.10 s (3H), 2.09 s (3H), 2.02 s (3H), 1.69 d (*J* = 1.1 Hz, 3H), 1.66 dd (*J* = 1.7, 6.8 Hz, 3H), 1.44 d (*J* = 7.1 Hz, 3H), 1.37 s (3H), 1.17 d (*J* = 7.1 Hz, 3H), 1.08 d (*J* = 6.6 Hz, 3H), 0.90 t (*J* = 6.1 Hz, 3H). HR-ESIMS *m*/*z* 1072.60225, 1086.60997, 1099.62465 (calcd for ^12^C_55_^13^CH_88_NaO_18_^+^ 1072.59019 (+11.24 ppm), ^12^C_56_^13^CH_90_NaO_18_^+^ 1086.60584 (+3.8 ppm), and C_58_H_92_NaO_18_^+^ 1099.61814 (+5.9 ppm)).

### 2.9. Compound **4** from **2**

^1^H NMR: *δ* 5.65 dd (*J* = 2.6, 9.8 Hz), 5.52 m, 5.51 d (*J* = 10.2 Hz), 5.37 m, 5.34 dd (*J* = 1.7, 9.1 Hz), 5.28 dt (*J* = 1.7, 10.5 Hz), 5.07 d (*J* = 10.9 Hz), 4.78 d (*J* = 6.3 Hz), 4.54 dd (*J* = 3.1, 11.7 Hz), 4.25 dd (*J* = 2.3, 8.1 Hz), 4.13 dd (*J* = 6.5, 11.3 Hz), 3.43 s (3H), 3.34 dq (*J* = 10.1, 5.7 Hz), 3.29 dq (*J* = 10.5, 7.2 Hz), 3.01 dd (*J* = 9.4, 18.6 Hz), 2.88 d (*J* = 9.2 Hz), 2.60 dd (*J* = 2.5, 18.6 Hz), 2.35–2.4 m, 2.32 (*J* = 1.9, 9.3 Hz), 2.22 s (3H), 2.10 s (3H), 2.09 s (3H), 2.02 s (3H), 1.70 d (*J* = 1.1 Hz, 3H), 1.62 dd (*J* = 1.7, 6.8 Hz, 3H), 1.44 d (*J* = 7.1 Hz, 3H), 1.37 s (3H), 1.17 d (*J* = 7.1 Hz, 3H), 1.08 d (*J* = 6.6 Hz, 3H), 0.90 t (*J* = 6.1 Hz, 3H). HR-ESIMS [M+Na^+^] *m*/*z* 1071.58258, 1085.60199, 1099.61165 (calcd for C_56_H_88_NaO_18_^+^ 1071.58629 (−4 ppm), C_57_H_90_NaO_18_^+^ 1085.60194 (−.5 ppm), and C_58_H_92_NaO_18_^+^ 1099.61759 (−5.9 ppm)).

### 2.10. Screening Process

NBT-T2 cells were purchased from Riken and used for cytotoxicity testing. NBT-T2 is a cell line derived from chemically induced rat bladder carcinoma cells [19]. The sponge extract was tested at 0.1, 1, and 10 *μ*g/mL in triplicate, while fractions were done at 0.01, 0.1, and 1 *μ*g/mL. The cells were cultured in Dulbecco's modified Eagle's medium (DMEM) supplemented with Sigma antibiotic-antimycotic, Biowest fetal bovine serum, Gibco MEM nonessential amino acid in a Falcon 24-well plate or 48-well plate. After adding the extract or a fraction, cells were incubated for 24 h under 5% CO_2_ at 36°C [16]. Then, the cells were observed under a microscope to evaluate viability of cells whether the fractions were cytotoxic or not.

### 2.11. MTT Assay

Cultured cells were inoculated to a 96-well plate with approximate cell density of 1 × 10^4^ cells/mL in DMEM. After 24 h incubation, a series of DMSO solution of compounds **1**–**3** were applied to each well and the final concentrations were adjusted as 0, 1, 12.5, 25, 37.5, 50, 62.5, to 75 ng/mL. Cells were incubated for another 24 h, and the media were replaced with 20 *μ*L of 5 g/mL MTT solution in PBS and incubated for 3.5 h. After removal of PBS solution, an amount of 150 *μ*L of DMSO was added to each well and the cells were reincubated for 15 min prior to measurement with a Tecan microplate reader at 590 nm with reference filter at 620 nm [20, 21].

## 3. Results and Discussion

**Figure 1:**
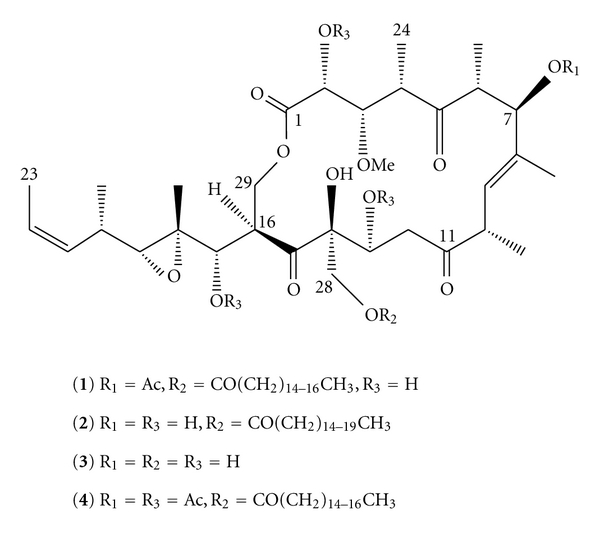
Structures of compounds (**1**)–(**4**).

As an EtOAc soluble portion of a methanolic extract of the sponge K09-02 showed potent cytotoxicity against cultured NBT-T2 cells, the portion was separated repetitively on a silica gel column followed by Si-60 HPLC affording three compounds **1**, **2**, and **3 **as shown in Figure 1. 

By inspecting ^1^H and ^13^C NMR spectra of compound **1** together with database search (Tables 1 and 2, Figures  S1 and  S2 (Supplementary Materials available online at doi:10.5402/2011/852619)), we could readily identify that it is a member of candidaspongiolide, a series of 18-membered cytotoxic macrolide retaining one of fatty acid moieties from C_14_ to C_18_ at C-28 [15]. HR-ESIMS of our material exhibited molecular-related ions at* m*/*z* 945.55514, 959.57079, 973.58884 [M+Na]^+^ indicating that compound **1** is candidaspongiolide esterified with the homologs of three saturated fatty acids (palmitic, margaric, and stearic acids). 

Compound **2** was obtained as a colorless glass with [*α*]_D_^25^ +72. After elucidation of its ^1^H and ^13^C NMR spectra, compound **2** was found to be an analog of **1**. However, the ^13^C NMR spectrum showed two carbonyl carbons at *δ*_C_ 171.3 q (C-1) and 173.1 (C-35) instead of three in **1** (Table 1, Figure  S3). As the signals for an acetoxy group (*δ*_H_ 2.04 s, *δ*_C_ 21.5 q) in **1** are missing in **2**, it was suggested that **2** is a deacetyl derivative of **1**. The lack of the acetyl group is in a good agreement with ^1^H NMR spectrum and COSY analysis showing that H-7 proton signal (*δ*_H_ 4.12 d, *J *= 10.0 Hz) in **2** shifted to higher field than that (*δ*_H_ 5.41 d, *J *= 10.7 Hz) in **1** (Table 2). HR-ESIMS of **2** showed a series of sodiated ions [M+Na]^+^ at *m/z* 903.54964, 917.56023, 931.57576, 945.60036, and 959.61056 corresponding to the presence of C_16_ to C_20_ esters. For structural confirmation, compound **2** was acetylated to give tetraacetate **4**, which showed signals identical with **4** obtained from **1 (**Figure  S4). Compound **4** exhibited four acetyl signals at *δ*_H_ 2.22 s, 2.10 s, 2.09 s, and 2.02 s and molecular-related ions corresponding to macrolide esters with C_16_ to C_18_ fatty acids. 

Compound **3 **was isolated as a yellowish glass with [*α*]_D_^25^ +97. Its molecular formula was established as C_32_H_50_O_13_ by observing a molecular-related ion at *m/z* 665.31522 [M+Na]^+^ in HR-ESIMS. ^1^H and ^13^C NMR spectra (Tables 1 and 2, Figures  S5 and  S6) revealed that compound **3** has a similar macrolide structure to that of compound **1** except for the lack of a fatty acid ester moiety and an acetate found in **1**. Higher field chemical shifts observed for H-7 (*δ*_H_ 4.03) and H-28 (*δ*_H_ 3.75) indicated that **3** is devoid of acyl groups. Close similarity of ^1^H and ^13^C NMR data of **3** to **1** (Table 2) indicated that the macrolide core structure of compound** 3 **is identical to compound **1**. 

All of natural compounds **1**–**3** exhibited potent cytotoxicity, IC_50_ 37, 4.7, and 19 ng/mL, against NBT-T2 cells. The result is not in good agreement with those reported by Meragelman and coworkers, that is, candidaspongiolide (**1**) showed stronger growth inhibition (GI_50 _14 ng/mL) than the core compound (42 ng/mL) [15]. Additionally Paul et al. paperd the importance of a linear carbon chain on the cytotoxicity in the case of amphidinol [22]. The difference may be explained either by the number of cell lines or by different sensitivity of NBT-T2 cells.

## Figures and Tables

**Table 1:** ^13^C NMR data for compounds **1**, **2**, and **3**.

C no.	1^15,a^	1^a^	2^a^	3^b^
1	171.3 qC	171.3 qC	171.3 qC	171.3 qC
2	70.7 CH	70.8 CH	70.8 CH	72.9 CH
3	83.4 CH	83.3 CH	83.1 CH	84.7 CH
4	47.8 CH	47.7 CH	47.9 CH	49.5 CH
5	214.6 qC	214.9 qC	216.2 qC	217.6 qC
6	48.5 CH	48.3 CH	49.8 CH	51.1 CH
7	80.1 CH	80.1 CH	79.2 CH	79.9 CH
8	131.6 qC	132.0 qC	136.2 qC	139.0 qC
9	132.0 CH	131.7 CH	129.4 CH	129.8 CH
10	46.2 CH	46.1 CH	45.9 CH	46.5 CH
11	211.5 qC	211.7 qC	211.9 qC	212.7 qC
12	42.6 CH_2_	42.7 CH_2_	42.5 CH_2_	44.3 CH_2_
13	68.9 CH	68.8 CH	69.0 CH	69.3 CH
14	81.6 qC	81.6 qC	81.6 qC	85.0 qC
15	210.8 qC	211.0 qC	211.2 qC	216.5 qC
16	46.9 CH	47.0 CH	46.9 CH	46.4 CH
17	77.8 CH	77.7 CH	77.7 CH	78.4 CH
18	62.7 qC	62.7 qC	62.6 qC	63.9 qC
19	67.1 CH	67.0 CH	67.0 CH	67.4CH
20	31.1 CH	31.1 CH	30.9 CH	32.4 CH
21	129.7 CH	129.4 CH	129.6 CH	131.6 CH
22	125.5 CH	125.5 CH	125.4 CH	126.2 CH
23	13.5 CH_3_	13.4 CH_3_	13.3 CH_3_	13.3 CH_3_
24	14.6 CH_3_	14.5 CH_3_	14.4 CH_3_	15.3 CH_3_
25	14.7 CH_3_	14.5 CH_3_	15.0 CH_3_	15.6 CH_3_
26	10.8 CH_3_	10.7 CH_3_	10.2 CH_3_	10.5 CH_3_
27	16.3 CH_3_	16.2 CH_3_	16.4 CH_3_	15.7 CH_3_
28	63.5 CH_2_	63.5 CH_2_	63.5 CH_2_	65.7 CH_2_
29	63.2 CH_2_	63.2 CH_2_	62.8 CH_2_	64.8 CH_2_
30	11.1 CH_3_	11.0 CH_3_	10.9 CH_3_	11.5 CH_3_
31	18.6 CH_3_	18.4 CH_3_	18.3 CH_3_	18.7 CH_3_
32	60.3 CH_3_	60.3 CH_3_	60.3 CH_3_	60.3 CH_3_
33	169.5 qC	169.9 qC	—	—
34	21.6 CH_3_	21.5 CH_3_	—	—
35	173.5 qC	173.7 qC	173.6 qC	—
36	34.2 CH_2_	34.1 CH_2_	34.0 CH_2_	—
37	29.0 CH_2_	29.0 CH_2_	29.1 CH_2_	—

^a^Measured in CDCl_3_. ^b^Measured in CD_3_OD.

**Table 2:** ^1^H NMR data for compounds **1**, **2**, and **3** (*J* in Hz).

C no.	1^15,a^	1^a^	2^a^	3^b^
1	—	—	—	—
2	3.96 dd (1.0, 7.3)	3.96 dd (1.3, 7.5)	3.98 dd (1.3, 7.8)	3.76 d (2.2)
3	3.64 dd (1.3, 7.8)	3.67 dd (1.3, 8.0)	3.67 dd (1.3, 8.4)	3.81 dd (2.2, 9.8)
4	3.12 m	3.13 dd (8.0, 7.1)	3.10 dq (8.4, 7.3)	3.16 dq (9.8, 7.1)
5	—	—	—	—
6	3.18 dq (10.7, 7.3)	3.22 dq (10.7, 6.8)	3.04 dq (9.8, 6.8)	3.16 dq (10.0, 7.1)
7	5.39 d (10.7)	5.41 d (10.7)	4.12 d (10.0)	4.03 d (10.0)
8	—	—	—	—
9	5.60 d (9.3)	5.62 d (9.6)	5.48 d (10.5)	5.33 d (9.3)
10	3.38 dq (9.3, 6.8)	3.41 dq (9.6, 7.0)	3.49 dq (10.5, 7.1)	3.36 dq (9.3, 6.8)
11	—	—	—	—
12	2.66 dd (9.8, 16.1)	2.69 dd (9.8, 16.1)	2.72 dd (9.8, 16.1)	2.75 dd (9.5, 17.6)
	2.49 dd (2.4, 16.1)	2.49 dd (2.5, 16.1)	2.51 dd (2.0, 16.1)	2.23 dd (2.0, 17.6)
13	4.40 m	4.42 m	4.39 dt (2.0, 9.8)	4.44 dd (2.9, 9.5)
14	—	—	—	—
15	—	—	—	—
16	4.02 dt (3.9, 10.9)	4.03 ddd (3.9, 10.5, 11.5)	4.09 dt (3.9, 11.0)	4.08 ddd (3.9, 10.8, 11.4)
17	3.12 m	3.13 m	3.20 dd (11.0)	3.20 d (10.7)
18	—	—	—	—
19	2.56 d (9.3)	2.59 d (9.3)	2.58 d (9.8)	2.62 d (9.3)
20	2.44 m	2.47 m	2.47 m	2.48 m
21	5.23 dt (1.5, 10.7)	5.25 ddd (0.7, 10.2, 10.9)	5.24 dt (1.5, 10.5)	5.31 m
22	5.48 dq (10.7, 6.8)	5.51 dq (10.9, 6.8)	5.49 dq (10.5, 6.8)	5.51 dq (10.7, 6.8)
23	1.59 dd (1.5, 6.8)	1.62 dd (1.2, 6.8)	1.62 dd (1.5, 6.8)	1.62 dd (1.7, 6.8)
24	1.18 d (6.8)	1.21 d (7.1)	1.21 d (7.3)	1.23 d (7.1)
25	1.13 d (7.3)	1.16 d (6.8)	1.28 d (6.8)	1.26 d (7.1)
26	1.54 brd (1.0)	1.59 d (0.8)	1.63 s	1.65 d (1.5)
27	1.07 d (6.8)	1.09 d (7.0)	1.10 d (7.1)	1.03 d (6.8)
28	4.44 d (11.7)	4.46 d (11.5)	4.45 d (11.5)	3.75 d (10.5)
	4.19 d (11.7)	4.22 d (11.5)	4.21 d (11.5)	3.76 d (10.5)
29	4.17 dd (3.7, 9.8)	4.20 dd (3.7, 10.2)	4.24 dd (3.0, 9.5)	4.35 dd (3.9, 10.5)
	4.10 dd (10.2, 10.9)	4.12 dd (10.2, 11.2)	4.08 m	3.91 dd (10.5, 11.4)
30	1.38 s	1.42 s	1.42 s	1.35 s
31	1.11 d (6.4)	1.13 d (6.3)	1.14 d (6.6)	1.11 d (6.6)
32	3.28 s	3.31 s	3.30 s	3.39 s
33	—	—	—	—
34	2.01 s	2.04 s	—	—
35	—	—	—	—
36	2.24 t (7.6)	2.27 t (7.6)	2.27 t (11.8)	
37	1.53 brs	1.59 brs	1.25 brs	
OH-2	2.85 d (7.3)	2.92 d (7.5)	2.99 d (7.8)	2.92 d (7.5)
OH-13	—		4.71 s	

^a^Measured in CDCl_3_. ^b^Measured in CD_3_OD.
